# Stockouts of HIV commodities in public health facilities in Kinshasa: Barriers to end HIV

**DOI:** 10.1371/journal.pone.0191294

**Published:** 2018-01-19

**Authors:** Tinne Gils, Claire Bossard, Kristien Verdonck, Philip Owiti, Ilse Casteels, Maria Mashako, Gilles Van Cutsem, Tom Ellman

**Affiliations:** 1 Médecins sans Frontières, Southern Africa Medical Unit, Cape Town, South Africa; 2 Institute of Tropical Medicine, Antwerp, Belgium; 3 Academic Model Providing Access to Healthcare, Eldoret, Kenya; 4 The International Union Against Tuberculosis and Lung Disease, Paris, France; 5 Médecins sans Frontières, Operational Centre Brussels, Kinshasa, DRC; Jagiellonian University, POLAND

## Abstract

Stockouts of HIV commodities increase the risk of treatment interruption, antiretroviral resistance, treatment failure, morbidity and mortality. The study objective was to assess the magnitude and duration of stockouts of HIV medicines and diagnostic tests in public facilities in Kinshasa, Democratic Republic of the Congo. This was a cross-sectional survey involving visits to facilities and warehouses in April and May 2015. All zonal warehouses, all public facilities with more than 200 patients on antiretroviral treatment (ART) (high-burden facilities) and a purposive sample of facilities with 200 or fewer patients (low-burden facilities) in Kinshasa were selected. We focused on three adult ART formulations, cotrimoxazole tablets, and HIV diagnostic tests. Availability of items was determined by physical check, while stockout duration until the day of the survey visit was verified with stock cards. In case of ART stockouts, we asked the pharmacist in charge what the facility coping strategy was for patients needing those medicines. The study included 28 high-burden facilities and 64 low-burden facilities, together serving around 22000 ART patients. During the study period, a national shortage of the newly introduced first-line regimen Tenofovir-Lamivudine-Efavirenz resulted in stockouts of this regimen in 56% of high-burden and 43% of low-burden facilities, lasting a median of 36 (interquartile range 29–90) and 44 days (interquartile range 24–90) until the day of the survey visit, respectively. Each of the other investigated commodities were found out of stock in at least two low-burden and two high-burden facilities. In 30/41 (73%) of stockout cases, the commodity was absent at the facility but present at the upstream warehouse. In 30/57 (54%) of ART stockout cases, patients did not receive any medicines. In some cases, patients were switched to different ART formulations or regimens. Stockouts of HIV commodities were common in the visited facilities. Introduction of new ART regimens needs additional planning.

## Introduction

Ending HIV will require resilient supply chains to ensure uninterrupted access to diagnostics and antiretroviral treatment (ART). Stockouts of testing kits and antiretroviral drugs (ARVs) may prevent countries from reaching the international 90-90-90 targets, i.e. for 90% of people living with HIV/AIDS (PLHIV) to know their HIV status, to put 90% of those diagnosed on ART and to keep the viral load suppressed in 90% of those on ART by 2020 [1,2]. Stockouts of ARVs lead to delays in treatment initiation, unstructured treatment interruptions, and disengagement of patients with care [3,4]. These situations have negative consequences for individuals and populations, as they increase the risk of opportunistic infections, treatment failure, viral resistance and death [5–9]. The World Health Organization (WHO) considers any stockout of a routinely used ARV in an ART dispensary over the course of a year an early warning indicator for ART resistance [10]. Supply disruptions of other HIV-commodities can also do a lot of damage: stockouts of diagnostic tests cause gaps in testing activities and delays in treatment initiation, while stockouts of cotrimoxazole interfere with successful prevention of opportunistic infections [11,12].

Most countries, including those in Sub-Saharan Africa, aim for universal HIV treatment and provide access to cheap, one-pill-a-day regimens for the majority of the PLHIV [2]. However, an evaluation by the WHO of 1703 ART clinics in 35 countries showed that ARV stockouts are frequent: 36% reported at least one stockout in a 12–month reporting period between 2005 and 2014 [10]. Stockouts appeared to be more frequent in Sub-Saharan African countries than in other global regions [10]. The West and Central African region has made slow progress towards the 90-90-90 targets, despite accounting for 1 in 3 AIDS related deaths globally [13]. The need for acceleration of the HIV/AIDs response in this region, including fixing supply chains, has been recognised by the United Nations (UN) at the 2016 General Assembly [14].

As a response to rising reporting of global shortages, WHO has passed a resolution calling to assess the nature and magnitude of stockouts and shortages and to install early warning systems for prevention of local and national stockouts [15]. Yet country-wide data of facility stockouts are often non-existent, not available in the public domain or biased by self-reporting of the health workers under evaluation [16].

In the Democratic Republic of the Congo (DRC), country-wide information about stockouts of HIV commodities is not available. Indications of severe interruptions in ART and cotrimoxazole supply have however been reported by patient groups in Kasa-Vubu and North-Kivu [17,18]. In Kinshasa, teams of Médecins sans Frontières (MSF) have provided missing medicines during stockouts. No previous study has looked at stockouts in facilities in Kinshasa in a systematic way.

This study thus aimed to assess the magnitude and duration of stockouts of HIV commodities in public facility dispensaries and zonal warehouses in the province of Kinshasa, and to describe coping mechanisms to deal with stockouts at the level of the facilities.

## Methods

### Design

This was a cross-sectional survey including data collected through visits to facility pharmacies and warehouses in Kinshasa in April and May 2015.

### Setting

The DRC is the largest country in Sub-Saharan Africa, with a population of over 75 million, of which 40% live in urban areas [19]. Countrywide, 370000 people are estimated to be HIV positive [20]. In 2015, national treatment coverage was 33%, and only 3% of patients on ART had a viral load test done. In the capital Kinshasa, which is also a province, the estimated HIV prevalence is 1.6%, and 37000 PLHIV were on ART by the end of 2015 [20].

### Supply chain

In the DRC, HIV commodities are almost exclusively funded and procured by international donor agencies such as the Global Fund for AIDS, Tuberculosis and Malaria (GFATM) and the United States President’s Emergency Plan for AIDS Relief (PEPFAR). Supply chains are highly fragmented, with multiple tiers and supply chains run by different actors [21]. The DRC National Medicine Supply Programme oversees the overall medicine supply system, while the National HIV department develops the national HIV treatment guidelines and forecasts demand of HIV commodities. At the time of the survey, this forecast was based on provincially compiled pharmacy consumption reports, the national ART patient database, as well as data gathered by GFATM and PEPFAR. Supply chains for HIV commodities were managed largely in parallel by the two major donor agencies and their multiple implementing partners through a two-tiered system (PEPFAR) and a three-tiered system (GFATM). At the time of the survey, many facilities received support from both agencies at the same time for different activities and supplies, leading to frequent overlap and gaps.

Each province has a provincial warehouse that is run as a semi-independent entity contracted by donors for storage and distribution of medicines to the different health zones. The province of Kinshasa has 35 health zones, each with a zonal warehouse serving health facilities. The maximum traveling time by car between any facility and the closest warehouse in Kinshasa is five hours, with some warehouses adjacent to facilities.

In January 2015, the country had started a transition to a new first line regimen, leading to national supply disruptions at the time of the study. The study timing was not deliberately planned during this period but it created an opportunity to document the impact of the transition on ART availability in the facilities.

### Study population

The target population were ART-providing facilities in Kinshasa and zonal warehouses. For the study, facilities were sampled based on health zone and number of PLHIV under care. We did not include the military and the police health zones. From each of the 33 remaining health zones, we selected all the facilities with more than 200 patients on ART, the largest facility with 100 to 200 patients and the largest one with fewer than 100 patients on ART (if existent). This purposive sampling of low burden facilities was done to include facilities of different sizes and serving as many patients as possible, while considering feasibility and budget. We also targeted the 33 zonal warehouses.

### Variables and operational definitions

The variables included were the point prevalence of stockouts of a set of HIV commodities in the visited sites. A stockout was defined as no physical availability of the selected commodity in the facility pharmacy at the time of the survey visit. A complete first-line ART regimens consist of three active ingredients. Sometimes, two or more active ingredients are combined into a single tablet (also called a fixed-dose combination) to reduce the pill burden and improve adherence [22]. We have written the fixed-dose-combinations as the names of the active ingredients separated with a hyphen, and we have written the formulations with only one active ingredient as separate words. We selected the following ARVs for analysis: the two fixed-dose-combinations for first-line adult treatment; Tenofovir-Lamivudine-Efavirenz (TDF-3TC-EFV) and Zidovudine-Lamivudine-Nevirapine (AZT-3TC-NVP), and the Tenofovir-Lamuvidine (TDF-3TC) combination tablets commonly used as a first-line regimen (with Nevirapine) or a second-line regimen (with Lopinavir/ritonavir). These regimens were selected because at least 90% of ART patients in Kinshasa are treated with one of the included ART formulations [23]. We also assessed the availability of tablets of cotrimoxazole (CTX) for adults, which contain a combination of the antibiotics sulfamethoxazole and trimethoprim, and of Determine™ HIV-1/2 rapid diagnostic tests (HIV test) [24]. In case of stockouts, we registered the duration until the day of the survey visit and, in cases of ART stockouts, the solutions the facility would propose to patients needing the unavailable ART. We differentiated between giving no ART at all (ask to return later, refer to another facility) and substitution with another formulation or regimen. High-burden facilities were those that reported more than 200 patients on ART and low-burden facilities those that reported 200 or fewer patients on ART in March 2015 according to the national database.

### Data sources

For the 33 included health zones, the number of patients on ART was retrieved from the national database [23]. For the facilities included in the study, the number of patients on ART was retrieved from the patient registers kept at the facilities. Outcome data were collected on two standard paper questionnaires—one for health facilities and one for warehouses—in April and May 2015. The questionnaires can be found under supporting information (S1 and S2 Texts). Study teams of two persons each were trained for two days on the use of the questionnaire, including test visits to two facilities per team. At arrival at the facility or warehouse, the teams administered the questionnaire to the person in charge of the pharmacy. The point prevalence of stockouts was determined through physical check of the availability of the items in the pharmacy shelves. Data collected from existing registers were used to determine the duration in days of a stockout up to the day of the survey visit. The facility coping practise in case of a stockout was recorded as reported by the responsible pharmacist. Senior medical MSF staff conducted supervision visits of the teams during the survey. The data used for this analysis consist of a selection of the originally collected data (S1 Dataset), including only the items that are most commonly used in the facilities.

### Statistical analysis

Data from the questionnaires were initially entered into Microsoft Excel 2015 by trained data clerks and then analysed with Stata software version 14 (*StataCorp*, *College Station*, *TX*, *USA*). Frequencies and percentages were used to describe categorical variables and medians with interquartile ranges for numerical variables.

### Ethics

The study fulfilled the exemption criteria set by the Ethics Review Board (ERB) of MSF, Geneva, Switzerland, for a-posteriori analyses of routinely collected data and thus did not require MSF ERB review. The study was also approved by the Ethics Advisory Group of the International Union Against Tuberculosis and Lung Disease, Paris, France. As this was a record review study, informed consent was not required. The study did not require ethical approval in country, but the protocol was approved by the provincial HIV programme of Kinshasa.

## Results

**Fig 1:**
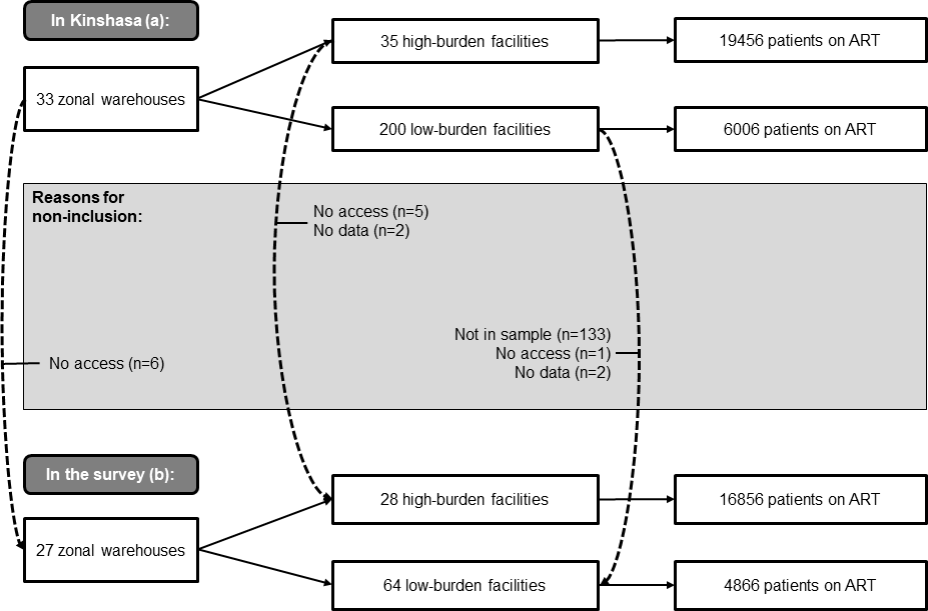
Selection of public health facilities and numbers of patients on ART, Kinshasa 2015. (a) Antiretroviral treatment (ART) facilities in Kinshasa. Out of 35 existing health zones in Kinshasa, the two zones managed by the police and the military, serving 1187 patients, were excluded before analysis. (b) ART facilities included in the study. All high-burden facilities were targeted, and a purposive sample of low-burden facilities, including for each health zone the largest facility with 100–200 patients and the largest facility with < 100 patients.

We obtained information from 92 facilities serving 82% of the 26649 ART patients registered in Kinshasa, or 85% of the cohort registered outside police and military zones. We included 28 of the 35 high-burden health facilities in Kinshasa (Fig 1). These were providing ART to 16856 PLHIV or 63% of registered patients on ART. We also included 64 of 200 low-burden health facilities (19/23 facilities with 100–200 patients and 45/177 facilities with <100 patients) which were treating 4866 PLHIV or 18% of registered ART patients. Finally, we evaluated 27 of the 33 selected zonal warehouses which stock commodities for those facilities in Kinshasa.

**Fig 2:**
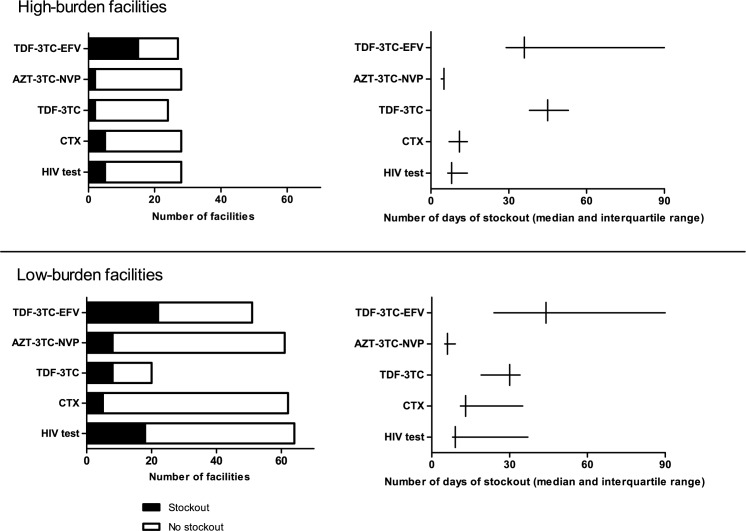
Number and duration of stockouts of HIV commodities in public health facilities, Kinshasa 2015. TDF-3TC-EFV: Tenofovir/Lamivudine/Efavirenz 300/300/600 mg tablets, 30 tablets. AZT-3TC-NVP: Zidovudine/Lamivudine/Nevirapine 300/150/200 mg tablets, 60 tablets. TDF-3TC: Tenofovir/Lamivudine 300/300 mg tablets, 30 tablets. CTX: Sulfamethoxazole/Trimethoprim 400/80 mg, tablets. HIV test: Determine^TM^ HIV-1/2 Test Kits.

The number and duration until the survey visit of stockouts of HIV commodities is shown in Fig 2. TDF-3TC-EFV was out of stock in 15 (56%) of 27 high-burden facilities and 22 (43%) of 51 low-burden facilities. The median duration of the TDF-3TC-EFV stockout was 36 days (interquartile range (IQR): 29–90) in high-burden facilities and 44 days (IQR: 24–90) in low-burden facilities. Of approximately 2500 patients on TDF-3TC-EFV, 1100 (44%) were followed at facilities experiencing stockouts during the study period. Stockouts of AZT-3TC-NVP were found in 2 (7%) out of 28 high-burden facilities and 8 (13%) out of 61 low-burden facilities. Of 14500 patients on AZT-3TC-NVP, 1300 patients (9%) were followed at the facilities experiencing stockouts. TDF-3TC was reported out of stock in 8% (2/24) of high-burden facilities, and in 40% (8/20) of the low-burden facilities.

CTX tablets were out of stock in 18% (5/28) of high-burden and 8% (5/62) of low-burden facilities. Out of 18500 patients registered on cotrimoxazole, 3200 (17%) were attending those facilities with a stockout. HIV test kits were not available in 5 (18%) out of 28 high-burden facilities and 18 (28%) of the 64 low-burden facilities.

**Fig 3:**
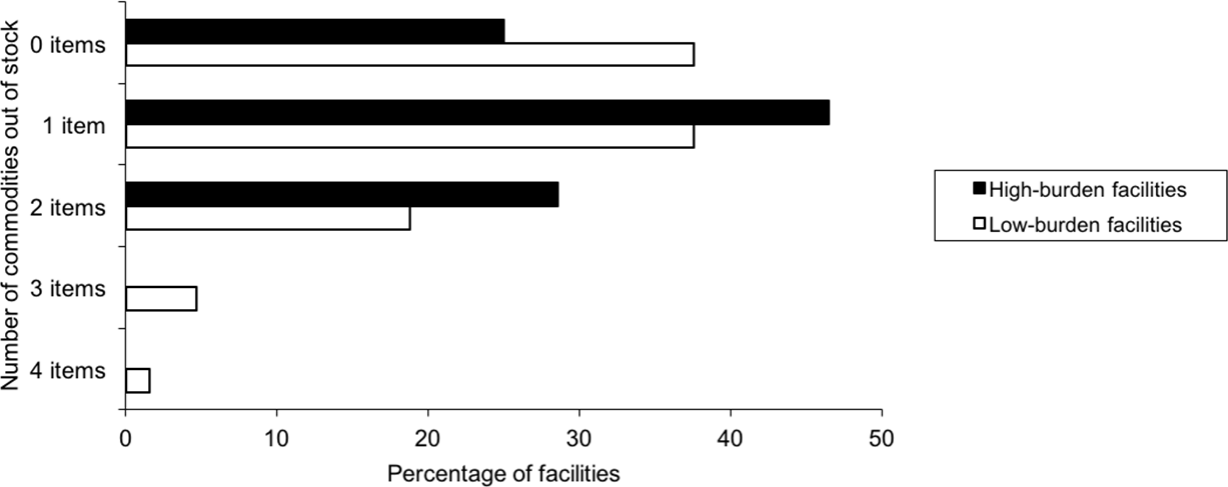
Percentage of facilities with number of HIV commodities out of stock, Kinshasa 2015.

Out of 28 high-burden facilities, 13 (46%) had 1 item out of stock and 8 (29%) had 2 stockouts (Fig 3). Out of 64 low-burden facilities, 24 (38%) had 1 stockout, 12 (19%) had 2 stockouts, 3 (5%) had 3 stockouts and one (2%) had 4 stockouts. 25% (7/28) of high-burden and 38% (24/64) of low-burden facilities had no stockouts. The facilities experiencing stockouts were serving 71% (15445/21722) of all patients attending included facilities.

For 41 (out of 45, 91%) stockout events of AZT-3TC-NVP, TDF-3TC, CTX or HIV tests in health facilities, we could check the associated zonal warehouse. In 30 (73%) of these stockout events, a product was absent at the facility but present at the warehouse during the same period. The median time between facility and warehouse visits was 6 days (IQR: 5–20).

Overall, in 30 out of 57 cases (54%) of ART stockouts, patients left the facility without any ART. In the case of TDF-3TC-EFV stockouts where ART was given (24/37), patients received another formulation (TDF-3TC with EFV, 18/24, 82%), or a substitution regimen (AZT-3TC-NVP, 4/24, 18%). In the remaining 7% (1/15) of high-burden and 55% (12/22) of low-burden facilities with TDF-3TC-EFV stockouts, no alternative ARV was given. In the case of AZT-3TC-NVP stockouts, the patients did not receive any ARV in all 10 facilities facing a stockout. In facilities with TDF-3TC stockouts, 2 out of 10 reported substituting the regimen with AZT-3TC-NVP, while 8 reported giving no ARVs.

Out of 92 facilities visited, 16% commonly provided one ARV (n = 15), 38% two ARVs (n = 35) and 46% all three surveyed ARVs (n = 42). Those facilities providing more ARVs where at higher risk of stockouts, but those with three ARVs were better placed to provide a substitution regimen. Out of those facilities commonly stocking only one ARV, 20% had a stockout (3/15) on the day of the survey visit, of which 1 (33%) managed to substitute the missing ARV. Among those facilities commonly providing two ARVs, 48% (17/35) had a stockout, of which 29% (5/17) provided a substitute. Among those commonly stocking three ARVs, 67% (28/42) had at least one ARV missing, out of which 68% (19/28) provided a substitution regimen.

## Discussion

The study indicates that stockouts of different HIV commodities are frequent in the included public health facilities in Kinshasa, with stockouts often lasting several days to weeks and affecting facilities serving varying numbers of patients. This is a problem for individual and public health because it puts more people at risk of HIV transmission and viral resistance and it increases morbidity and mortality, particularly in patients with low immunity [6,7].

When stockouts occurred, the staff at the facilities substituted the missing ARV with other ARV formulations or regimens, referred patients to other centres, or asked them to return later. Similar coping strategies have been described in response to ART stockouts in Tanzania, leading to increased cost for patients, higher risk of treatment interruption and emergence of resistant strains [25]. TDF stockouts occurring in 2012 in South Africa led to initiation of patients with less optimal regimens, patients having to return more frequently and an increase in missed drug pick-ups [26].

Side effects due to TDF-3TC-EFV are less frequent and the pill burden is lighter as compared to AZT-3TC-NVP [27]. Substitution between the two regimens may lead to negative clinical consequences for patients [6]. Substituting drugs might also give a false sense of supply security, because even if patients continue on ART, the change does interfere with the supply of the alternative drugs. Giving other ARV in case of absence of TDF-3TC-EFV may have contributed to stockouts of TDF-3TC and AZT-3TC-NVP.

This study has several limitations. First, the stockouts were measured on the day of the survey visit, which produces a point estimate and does not reflect an average stockout rate. In case of a stockout, we used stock cards to estimate stockout duration, but this duration could only be measured until the visit date, and stockouts might have lasted longer. Also, when commodities were available, their expiry date was not verified as part of the study. This means that even when available, some items might have not been usable. A second limitation is the fact that the included low-burden facilities were not randomly sampled from the facilities in the province of Kinshasa, leading to selection bias. Thus, even if approximately 82% of the patients on ART in Kinshasa received treatment in the included facilities, it cannot be assumed that this survey is representative for Kinshasa. This is illustrated by the median [IQR] patient burden, which in high burden facilities is 429 [287–670] in the population and 450 [317–686] in the selection. However, in low-burden facilities the median patient burden is 4 [10–37] in the population and 61 [27–132] in the study selection. The included low-burden facilities, serving a higher number of patients than the median, are likely to commonly stock more different ARV regimens. In line with our findings, these facilities could thus have been at a higher risk of a stockout, but better equipped to give a substitution regimen. Third, aside from supply problems resulting from the change of first-line regimen, the survey did not evaluate the causes of stockouts or the drivers of coping mechanisms in different facilities. Similarly, the survey did not assess the impact of the stockouts on patients’ lack of adherence and clinical outcomes. Finally, the study coincided with the transition from AZT-3TC-NVP to TDF-3TC-EFV as the first-line ARV regimen of choice in the DRC. The initial supply of TDF-3TC-EFV early 2015 was meant to treat only pregnant and breastfeeding women, as decided by the National HIV department. Lack of communication led facilities to switch more patients, leading in turn to a national stockout which lasted until December 2015.

The survey results suggest that two types of stockouts occurred in the facilities during the analysis: stockouts of an item for which there were national shortages and stockouts of items available in country. Due to the national shortage of TDF-3TC-EFV, it is not possible to draw conclusions on availability of this regimen under routine conditions. A similar cross-sectional study in Kinshasa from 2016 showed that TDF-3TC-EFV was available in a larger proportion of facilities after the national shortages had been corrected [28]. Only 2% (1/43) of high burden facilities and 10% (5/50) of low burden ones had a stockout of the first-line regimen on the visit day. 10% (4/39) of high-burden and 6% (2/36) of low-burden facilities were out of stock of AZT-3TC-NVP. 16% (5/31) and 23% (3/13) of high-and low-burden facilities respectively had stockouts of TDF-3TC on the visit day. The comparison suggests that the national shortages in 2015 led to increased stockouts in facilities, but that ART stockouts still occur when there are no national stock problems.

Implementation of guidelines changes, like the introduction of TDF-3TC-EFV are likely to happen every few years, as evidence for better treatment becomes available. Supply chain problems at scale-up and decentralisation of ART have been documented before [29]. In South Africa, facility stockouts also increased during introduction of TDF-3TC-EFV as a first-line regimen in 2013 and there was a national stockout of second-line regimens in 2015 due to unanticipated scale-up [30]. As such critical periods for the management of supply chains will continue to occur at introduction of new ART regimens, the need for engagement of all actors, extensive supply planning and firm orders has been suggested [31, 32]. In DRC, introduction of rapidly changing WHO recommendations for superior drug regimens needs to be accompanied by similar preparation.

The second type of stockouts observed in 2015 were of items that were available at national level and even frequently in a nearby warehouse, but absent in the facility. While the national stockout during this period led to increased facility stockouts of TDF-3TC-EFV and the substituting regimens, the high proportions of stockouts of test kits and CTX indicate a structural, systematic problem in supply chain. These findings show that medicines often do not reach patients due to problems in the final part of the supply chain, despite the short distances between facilities and warehouses in Kinshasa. In the 2016 analysis, most items were stocked out less often than in 2015. However, not one of the investigated items was available in all high-or low-burden facilities, indicating ongoing supply challenges due to chronic and structural supply chain problems.

The lack of data from the last level in the supply chain has contributed to a neglect of attention to the last mile and denial of stockout existence in many countries. In South Africa stockouts are reported by citizens through a free telephone number and traced up the supply chain, with all cases published on a public website [30, 33]. This has contributed to greater attention to the supply chain by government and improved transparency and quality control. Similar civil society engagement in DRC, with funding for promotion and tools, like a free phone number, might be valuable [34].

Actions are also necessary to ensure ‘last mile’ delivery. Simplifying the supply chain by reducing the tiers, installing informed push systems or direct delivery to the last mile have shown benefit in other settings [35–37]. Information technology can be used to report stock levels in real time to assist supply planning and provide early warning for stockouts [16, 38–39]. Clinical and supply guidance from country programmes should inform facilities on best coping mechanisms in case of stockouts to prevent treatment interruption and suboptimal treatment.

As pointed out by Bogaert et al., the lack of publicly available data on stockouts and problems surrounding definitions of stockouts and shortages have led to limited global quantification of stockouts and the identification of their causes. In that qualitative study, European stakeholders identified manufacturing, economic, supply and distribution problems, as the main causes of stockouts [40]. The WHA69.25 resolution “Addressing the Global Shortage of Medicines and Vaccines”, which followed increased global reporting of drug shortages, echoed those challenges [15]. A report commissioned by the European Association of Euro-Pharmaceutical Companies distinguished between predictable and unpredictable causes of stockouts [41]. In the DRC, predictable, mainly logistical challenges like just-in-time inventories, poor forecasting and transport problems, seem to be the most important drivers explaining stockouts [21]. On top of that, more than in most developing countries, medicines stockouts are exacerbated by unpredictable causes such as political unrest, financial crisis and recurrent epidemics [42]. Creation of reliable in-country supply chains has been recognised by the UN as a priority to reach 90-90-90 targets in West-and Central Africa [14].

At the time of this survey, no internationally recognised definition of drug stockouts existed and we based ourselves on the WHO definition for stockouts of ART as early warning indicators for drug resistance [10]. The definition used for this survey is consistent with the patient-focussed definition for stockouts resulting from a WHO convened stakeholder meeting in response to WHA69.25: “The complete absence of the medicine, health product or vaccine at the point of service delivery to the patient”[43].

The results of this study have been presented at national multi-stakeholder meetings. The data and additional analyses by the ministry of health and its partners have led to proposals for corrective actions. In the second half of 2015, the two main donors (GFATM and PEPFAR) reallocated the different health zones of Kinshasa for support by only one donor at a time, but optimal ongoing coordination will be necessary to avoid duplication and gaps. Due to the limitation of this cross-sectional design, repeated analysis and regular monitoring are needed to describe trends for the stockouts unrelated to the national shortage and to monitor management of future regimen introductions. Further research is also needed to assess the situation in more remote parts of the country.

This study provides quantitative data on stockouts of HIV commodities occurring in the field, where the last mile is neglected and where transition from one to another ARV regimen puts an additional strain on supply systems. In the DRC, like in other countries in West-and Central Africa, supply chains need fixing to catch up with global targets to end HIV.

## Supporting information

S1 Dataset Stockout survey: Aggregated responses (French). (DTA)

S1 Text Stockout questionnaire for facilities (French). (PDF)

S2 Text Stockout questionnaire for warehouses (French). (PDF)
