# Supplementary material for: Stockouts of HIV commodities in public health facilities in Kinshasa: Barriers to end HIV
Source: PLoS One. 2018 Jan 19;13(1):e0191294. doi: 10.1371/journal.pone.0191294 (PMC5774776; doi:10.1371/journal.pone.0191294)
Supplement: S1 Text — (PDF) [file pone.0191294.s002.pdf]

Questionnaire Formations Sanitaires

Avant de commencer, informez l'infirmier titulaire et présentez-vous avec la lettre, en expliquant que ;

- Médecins Sans Frontières (MSF) fait un travail d'enquête avec le Programme Provincial de la Lutte Contre le SIDA (PNLS) pour analyser la disponibilité des médicaments et des tests de dépistage dans les Formations Sanitaires (FOSA).
- L'objectif de l'enquête est de caractériser la situation et d'identifier les problèmes pour mieux y répondre. Des données sur les stock de médicaments seront collectées dans le but d'améliorer la situation.
- Vous aimeriez voir le stock des médicaments et poser quelques questions au responsable de la pharmacie.

Date (jj/mm/aaaa) :
Nom de la FOSA:

1iere PARTIE : ARV/CTX, Jour même

Réponses à remplir par médicament et par question dans la table 1

1. Combien de patients actifs prennent ces médicaments TARV dans votre structure ?
   *Demander de regarder le dernier rapport mensuel, spécifier le mois du rapport*____________

Continuer les questions seulement pour les médicaments qui sont utilisés dans la FOSA

2. Quelle est la consommation mensuelle rapportée de ces médicaments dans votre FOSA ?
   *Demander de regarder le dernier rapport mensuel, spécifier le mois du rapport*______________
3. Est-ce qu'il y a une rupture d'un de ces médicaments **aujourd'hui** ? *Ecrire oui ou non*
   Contrôlez les stocks ensemble.

Si la réponse est oui pour un certain médicament, continuer avec les questions suivantes.

4. Depuis combien de jours est-ce que le médicament n'a pas été disponible?
   a. Nombre de jours précis connu (spécifier)
   b. <une semaine
   c. Entre 1-2 semaines
   d. Entre 2 semaines et un mois
   e. > un mois
5. Quelle était la solution proposée aux patients pendant la rupture ?
   a. Rien donné, demandé aux patients de rentrer un/plusieurs jours plus tard
   b. Rien donné, référé à une autre FOSA
   c. Donné le bon médicament mais pour < 1 mois (spécifier combien de jours)
   d. Changement de dose, en (spécifier) *ex. EFV 200mg vs EFV 600mg*
   e. Changement de formulation (spécifier) *ex. Lop/rit sirop vs compr.*
   f. Changement posologie (spécifier) *ex. TDF/3TC + EFV vs TDF/3TC/EFV*
   g. Changement de régime en (spécifier) *ex. AZT/3TC vs ABC/3TC*
   h. Changement en bi ou monothérapie vs trithérapie en (spécifier)

	Table 1	Réponse à la question numéro				
	**Nom du médicament, dosage et formulation**	**1**	**2**	**3**	**4**	**5**
**ARV ADULTES**	EFAVIRENZ/LAMIVUDINE/TENOFOVIR, **EFV/3TC/TDF** 600/300/300mg, comprimées					
	LAMIVUDINE/ZIDOVUDINE, **3TC/AZT**, 150/300mg, comprimées					
	LAMIVUDINE/ZIDOVUDINE/NEVIRAPINE, **3TC/AZT/NVP**, 150/300/200mg, comprimées					
	LOPINAVIR/RITONAVIR, **LPV/R** 200/50mg, comprimées					
	TENOFOVIR/LAMIVUDINE, **TDF/3TC,** 300/300mg, comprimées					
**ARV PEDIATRIQUES**	LAMIVUDINE/ZIDOVUDINE, **3TC/AZT,** 30/60mg, comprimées					
	LAMIVUDINE/ZIDOVUDINE/NEVIRAPINE **3TC/AZT/NVP,** 30/60/50 mg, comprimées					
	EFAVIRENZ, **EFV,** 200mg, comprimées					
	LOPINAVIR/RITONAVIR, **LPV/R** 100/25mg, comprimées					
	LOPINAVIR/RITONAVIR, **LPV/R** 80/20mg/ml, 60ml, sirop					
	NEVIRAPINE, **NVP** 50mg, comprimées pour suspension oral **OU** 10mg/ml sirop					
**OI**	COTRIMOXAZOLE 480mg, comprimées					

2[ieme] PARTIE: ARV/CTX, 3 mois avant

Réponses à remplir par médicament et par question dans la table 2

6. Est-ce qu'il y a eu rupture d'un de ces médicaments **entre début janvier et fin mars 2015** ? *Ecrire oui ou non*. *Contrôlez les fiches de stocks ensemble.*

Si la réponse est oui pour un certain médicament, continuer avec les questions suivantes.

7. Si la réponse est oui, pour combien de jours ?
   a. Nombre de jours précis connu (spécifier)
   b. <une semaine
   c. Entre 1-2 semaines
   d. Entre 2 semaines et un mois
   e. > un mois
8. Quelle était la solution proposée pour les patients en ce moment ?
   f. Rien donné, demandé aux patients de rentrer un/plusieurs jours plus tard
   g. Rien donné, référé à une autre FOSA
   h. Donné bon médicament mais pour < 1 mois (spécifier combien de jours)
   i. Changement de dose, en (spécifier) *ex. EFV 200mg vs EFV 600mg*
   j. Changement de formulation (spécifier) *ex. Lop/rit sirop vs compr.*
   k. Changement posologie (spécifier) *ex. TDF/3TC + EFV vs TDF/3TC/EFV*
   l. Changement de régime en (spécifier) *ex. AZT/3TC vs ABC/3TC*
   m. Changement en bi ou monothérapie vs trithérapie en (spécifier)

	**Table 2**	**Réponse à la question numéro**		
	**Nom du médicament, dosage et formulation**	**6**	**7**	**8**
**ARV ADULTS**	EFAVIRENZ/LAMIVUDINE/TENOFOVIR, **EFV/3TC/TDF** 600/300/300mg, comprimées			
	LAMIVUDINE/ZIDOVUDINE, **3TC/AZT**, 150/300mg, comprimées			
	LAMIVUDINE/ZIDOVUDINE/NEVIRAPINE, **3TC/AZT/NVP**, 150/300/200mg, comprimées			
	LOPINAVIR/RITONAVIR, **LPV/R** 200/50mg, comprimées			
	TENOFOVIR/LAMIVUDINE, **TDF/3TC,** 300/300mg, comprimées			
**ARV PEDIATRIQES**	LAMIVUDINE/ZIDOVUDINE, **3TC/AZT,** 30/60mg, comprimées			
	LAMIVUDINE/ZIDOVUDINE/NEVIRAPINE, **3TC/AZT/NVP,** 30/60/50 mg, comprimées			
	EFAVIRENZ, **EFV,** 200mg, comprimées			
	LOPINAVIR/RITONAVIR, **LPV/R** 100/25mg, comprimées			
	LOPINAVIR/RITONAVIR, **LPV/R** 80/20mg/ml, 60ml, sirop			
	NEVIRAPINE, **NVP** 50mg, comprimées pour suspension oral OU 10mg/ml, sirop			
**OI**	COTRIMOXAZOLE 480mg, comprimées			

3ieme PARTIE : Dépistage

Réponses à remplir par question dans la table 3

9. Combien de tests de dépistage avez-vous fait le mois dernier?

*Demander de regarder ensemble le dernier rapport mensuel, spécifier le mois du rapport*___________________

10. Quelle est la consommation mensuelle rapportée du test DETERMINE dans votre FOSA ?

*Demander de regarder le dernier rapport mensuel ensemble, spécifier le mois du rapport*___________________

11. Est-ce qu'il y a une rupture de tests DETERMINE **aujourd'hui**? *Ecrire oui ou non*. *Contrôlez les stocks ensemble.*
12. Si la réponse est oui, depuis combien de jours ?
    a. Nombre de jours précis connu (spécifier)
    b. <une semaine
    c. Entre 1-2 semaines
    d. Entre 2 semaines et un mois
    e. > un mois
13. Est-ce vous vous rappelez s'il y a eu une rupture de tests DETERMINE **entre début janvier et fin mars 2015** ? *Ecrire oui ou non*. *Contrôlez les fiches de stocks ensemble*
14. Si la réponse est oui, pour combien de jours ?
    f. Nombre de jours précis connu (spécifier)
    g. <une semaine
    h. Entre 1-2 semaines
    i. Entre 2 semaines et un mois
    j. > un mois

**Table 3**	**Réponse à la question numéro**					
**Nom du test**	**9**	**10**	**11**	**12**	**13**	**14**
HIV 1+2, DETERMINE, test						

Merci pour votre temps et participation pour cette enquête !
