# Supplementary material for: Stockouts of HIV commodities in public health facilities in Kinshasa: Barriers to end HIV
Source: PLoS One. 2018 Jan 19;13(1):e0191294. doi: 10.1371/journal.pone.0191294 (PMC5774776; doi:10.1371/journal.pone.0191294)
Supplement: S2 Text — (PDF) [file pone.0191294.s003.pdf]

Questionnaire Dépôts des Zones de Santé

Avant de commencer, informez l'infirmier titulaire et présentez-vous avec la lettre, en expliquant que ;

- Médecins Sans Frontières (MSF) fait un travail d'enquête avec le Programme Provincial de la Lutte Contre le SIDA (PNLS) pour analyser la disponibilité des médicaments et des tests de dépistage dans les Formations Sanitaires (FOSA).
- L'objectif de l'enquête est de caractériser la situation et d'identifier les problèmes pour mieux y répondre. Des données sur les stocks de médicaments seront collectées dans le but d'améliorer la situation.
- Vous aimeriez voir le stock des médicaments et poser quelques questions au responsable de la pharmacie.

Date (jj/mm/aaaa) :
Nom de la ZdS:

1iere PARTIE : ARV/CTX, Jour même

Réponses à remplir par médicament et par question dans la table 1

---

1. Combien de patients actifs prennent ces médicaments TARV dans votre Zone ?
   *Demander de regarder le dernier rapport mensuel ensemble, spécifier le mois du rapport*____________

Continuer les questions seulement pour ces médicaments qui sont utilisés dans la FOSA

2. Quelle est la consommation mensuelle rapportée de ces médicaments dans votre Zone ?
   *Demander de regarder le dernier rapport mensuel ensemble, spécifier le mois du rapport*______________
3. Est-ce qu'il y a rupture d'un de ces médicaments **aujourd'hui** ? *Ecrire oui ou non*
   Contrôlez les stocks ensemble.

Si la réponse est oui pour un certain médicament, continuer avec les questions suivantes.

4. Depuis combien de jours est-ce que le médicament n'a pas été disponible?
   a. Nombre de jours précis connu (spécifier)
   b. <une semaine
   c. Entre 1-2 semaines
   d. Entre 2 semaines et un mois
   e. > un mois

	**Table 1**	**Réponse à la question numéro**			
	**Nom du médicament, dosage et formulation**	**1**	**2**	**3**	**4**
**ARV ADULTES**	EFAVIRENZ/LAMIVUDINE/TENOFOVIR, **EFV/3TC/TDF** 600/300/300mg, comprimées				
	LAMIVUDINE/ZIDOVUDINE, **3TC/AZT**, 150/300mg, comprimées				
	LAMIVUDINE/ZIDOVUDINE/NEVIRAPINE, **3TC/AZT/NVP**, 150/300/200mg, comprimées				
	LOPINAVIR/RITONAVIR, **LPV/R** 200/50mg, comprimées				
	TENOFOVIR/LAMIVUDINE, **TDF/3TC,** 300/300mg, comprimées				
**ARV PEDIATRIQES**	LAMIVUDINE/ZIDOVUDINE, **3TC/AZT,** 30/60mg, comprimées				
	LAMIVUDINE/ZIDOVUDINE/NEVIRAPINE **3TC/AZT/NVP,** 30/60/50 mg, comprimées				
	EFAVIRENZ, **EFV,** 200mg, comprimées				
	LOPINAVIR/RITONAVIR, **LPV/R** 100/25mg, comprimées				
	LOPINAVIR/RITONAVIR, **LPV/R** 80/20mg/ml, 60ml, sirop				
	NEVIRAPINE, **NVP** 50mg, comprimées pour suspension oral **OU** 10mg/ml sirop				
**OI**	COTRIMOXAZOLE 480mg, comprimées				

2ieme PARTIE: ARV/CTX, 3 mois avant

Réponses à remplir par médicament et par question dans la table 2

5. Est-ce qu'il y a eu rupture d'un de ces médicaments **entre début janvier et fin mars 2015** ? *Ecrire oui ou non*. C*ontrôlez les fiches de stocks ensemble*

Si la réponse est oui pour un certain médicament, continuer avec les autres questions.

6. Si la réponse est oui, pour combien de jours ?
    a. Nombre de jours précis connu (spécifier)
    b. <une semaine
    c. Entre 1-2 semaines
    d. Entre 2 semaines et un mois
    e. > un mois

	**Table 2**	**Réponse à la question numéro**	
	**Nom du médicament, dosage et formulation**	**5**	**6**
**ARV ADULTS**	EFAVIRENZ/LAMIVUDINE/TENOFOVIR, **EFV/3TC/TDF** 600/300/300mg, comprimées		
	LAMIVUDINE/ZIDOVUDINE, **3TC/AZT**, 150/300mg, comprimées		
	LAMIVUDINE/ZIDOVUDINE/NEVIRAPINE, **3TC/AZT/NVP**, 150/300/200mg, comprimées		
	LOPINAVIR/RITONAVIR, **LPV/R** 200/50mg, comprimées		
	TENOFOVIR/LAMIVUDINE, **TDF/3TC**, 300/300mg, comprimées		
**ARV PEDIATRIQES**	LAMIVUDINE/ZIDOVUDINE, **3TC/AZT,** 30/60mg, comprimées		
	LAMIVUDINE/ZIDOVUDINE/NEVIRAPINE, **3TC/AZT/NVP,** 30/60/50 mg, comprimées		
	EFAVIRENZ, **EFV,** 200mg, comprimées		
	LOPINAVIR/RITONAVIR, **LPV/R** 100/25mg, comprimées		
	LOPINAVIR/RITONAVIR, **LPV/R** 80/20mg/ml, 60ml, sirop		
	NEVIRAPINE, **NVP** 50mg, comprimées pour suspension oral **OU** 10mg/ml, sirop		
**OI**	COTRIMOXAZOLE 480mg, comprimées		

3^{ieme} PARTIE : Dépistage

Réponses à remplir par question dans la table 3

7. Combien de tests de dépistage avez-vous fait le mois dernier?

*Demander de regarder ensemble le dernier rapport mensuel, spécifier le mois du rapport*___________________

8. Est-ce qu'il y a rupture de tests DETERMINE **aujourd'hui**? *Ecrire oui ou non*. *Contrôlez les stocks ensemble.*
9. Si la réponse est oui, depuis combien de jours ?
   a. Nombre de jours précis connu (spécifier)
   b. <une semaine
   c. Entre 1-2 semaines
   d. Entre 2 semaines et un mois
   e. > un mois
10. Est-ce vous vous rappelez s'il y a eu rupture de tests DETERMINE **entre début janvier et fin mars 2015** ? *Ecrire oui ou non*. *Contrôlez les fiches de stocks ensemble*
11. Si la réponse est oui, pour combien de jours ?
    f. Nombre de jours précis connu (spécifier)
    g. <une semaine
    h. Entre 1-2 semaines
    i. Entre 2 semaines et un mois
    j. > un mois

**Table 3**	**Réponse à la question numéro**				
**Nom du test**	**7**	**8**	**9**	**10**	**11**
HIV 1+2, DETERMINE, test					

Merci pour votre temps et participation pour cette enquête !
